# Labia Majora Rejuvenation With Hybrid Filler: A Narrative Review of the Literature and Report of Two Cases

**DOI:** 10.1111/jocd.70074

**Published:** 2025-02-27

**Authors:** Andrea Acevedo, Andrea Marcela Parra, Andreina Martinez Amado, Valentina Dicker, Desiree Castelanich, Lina Velasquez, Luis Alberto Parra

**Affiliations:** ^1^ Aesthetic Medicne University Del Rosario Cali Colombia; ^2^ Oculoplastic Surgery‐ Ophthalmologist Sociedad Internacional De Rejuvenecimiento Facial no Quirúrgico (SIRF) Barranquilla Colombia; ^3^ Aesthetic Medicine, Clinical Research Universidad De Salamanca Bogota Colombia; ^4^ Aesthetic Medicne University Del Rosario Bogota Colombia; ^5^ Dermatologist Sociedad Argentina De Dermatologia Buenos Aires Argentina; ^6^ Dermatologist Sociedad Colombiana De Dermatología Cali Colombia; ^7^ Aesthetics Medicine Sociedad Internacional De Rejuvenecimiento Facial no Quirúrgico (SIRF) Barranquilla Colombia

**Keywords:** calcium hydroxyapatite, case report, hyaluronic acid, hybrid filler, labia majora, vaginal

## Abstract

**Background:**

Esthetic gynecology addresses the growing demand for treatments covering functional and esthetic concerns of the external genitalia, significantly impacting women's confidence and sexual relationships. While hyaluronic acid (HA) and calcium hydroxylapatite (CaHA) have shown promise in vaginal rejuvenation, data on hybrid filler applications remain limited.

**Objective:**

This narrative review examines the anatomical, structural, and psychological aspects of labia majora rejuvenation, outlining available treatment options and presenting the authors' experience with a novel HA/CaHA hybrid filler.

**Methods:**

This paper combined a narrative literature review using online databases PubMed and Google Scholar, between 2015 and 2024, with the keywords: “labia majora,” “rejuvenation,” “aesthetic treatments,” and “fillers” with a case series of two patients treated with a hybrid CaHA/HA (Radiesse and Belotero Balance Merz Pharmaceuticals GmbH) filler for labia majora volume loss and laxity.

**Results:**

Sixty‐day follow‐up evaluations by four experienced physicians revealed substantial improvements in labia majora esthetics. A marked improvement in skin coloration was observed in both patients, with skin appearing more even and less hyperpigmented. Both patients showed noticeable volume restoration, resulting in a fuller appearance of the labia majora. Overall aesthetic improvement was evident, as confirmed by the patients' perception. Patient‐reported genital appearance satisfaction, as measured by the GAS score, improved by a mean of 41% (pretreatment mean: 28/33; posttreatment mean: 15/33).

**Conclusion:**

These two case reports and a narrative literature review suggest that a combined HA/CaHA filler may be a safe and effective treatment for labia majora rejuvenation.

## 1 Introduction

The pursuit of ideal body image is increasingly influencing women's attention to vaginal and vulvar rejuvenation as they age [1, 2]. Cosmetic gynecology now addresses esthetic concerns (pigmentary disorders, vaginal atrophy, and laxity) and functional issues (sexual well‐being, quality of life), significantly impacting self‐esteem and overall well‐being [2, 3]. This growing field is attracting attention from cosmetic dermatology, esthetic medicine, and plastic surgery, leading to the exploration of various nonsurgical treatments, including chemical peels, platelet‐rich plasma, laser therapy, radiofrequency, and injectable fillers such as hyaluronic acid and calcium hydroxyapatite [4, 5, 6]. However, the widespread adoption of these treatments is limited by technical and anatomical complexities. The 2023 ISAPS annual survey reported a 19.6% increase in female genital cosmetic surgery for vaginal rejuvenation and a 14.8% increase in labiaplasty procedures compared to 2022 [7]. This narrative review, focusing on nonsurgical approaches, examines the anatomical, structural, and psychological aspects of labia majora rejuvenation using injectable fillers. A narrative literature search was performed in PubMed and Google Scholar and Embase (January 1, 2015—December 31, 2024) using the keywords: “labia majora,” “rejuvenation,” “vaginal,” “aesthetic gynecology,” “hyaluronic acid,” “calcium hydroxylapatite,” and “dermal filler.” The search strategy employed Boolean operators (AND, OR) to optimize results. Studies were included if they focused on nonsurgical labia majora rejuvenation using injectable fillers, were published in English, and contained at least 10 participants. Studies on surgical techniques and those not published in peer‐reviewed journals were excluded.

### 1.1 Anatomy of Female External Genital

**FIGURE 1:**
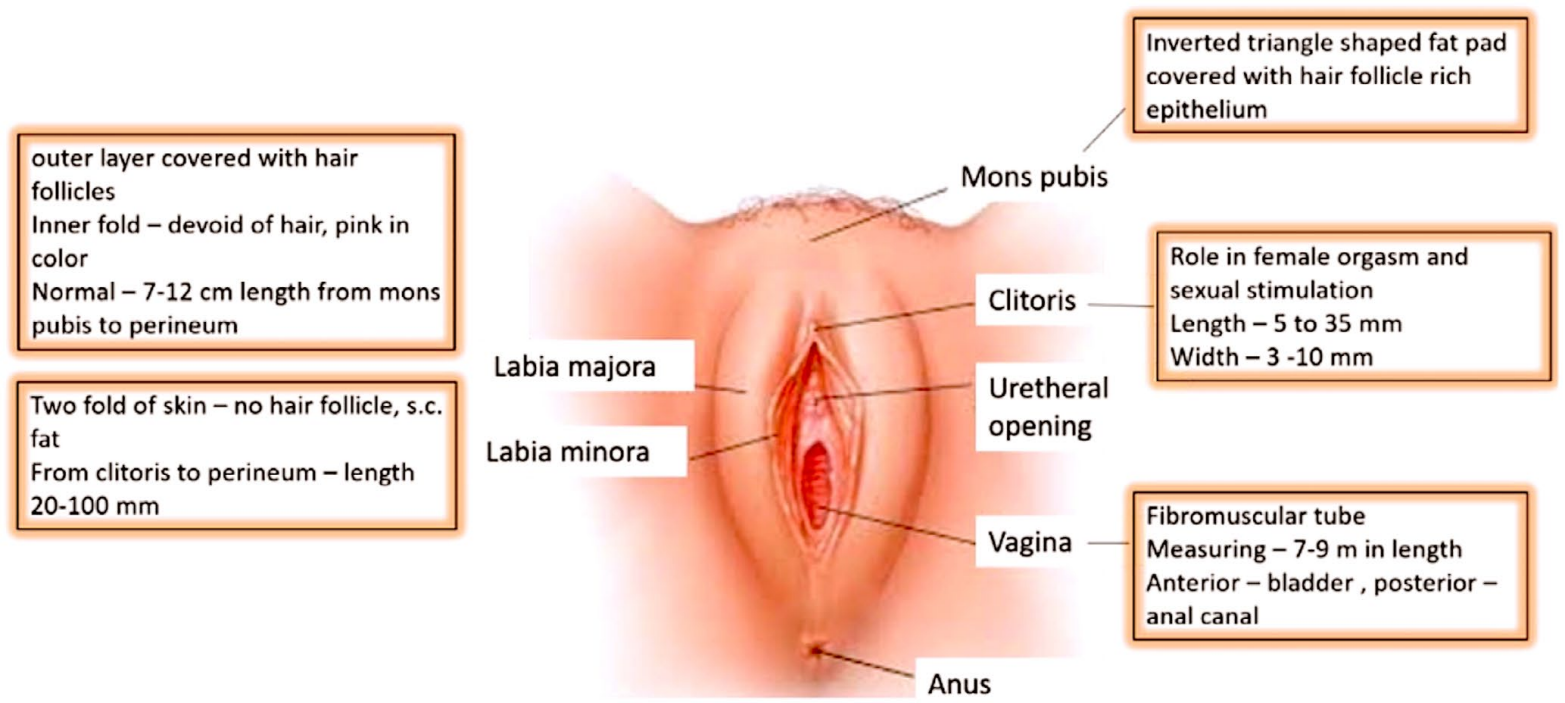
Diagram highlighting important features of the external genitalia in the box. Adapted from [3]. Image used with authorization: J Cosmet Dermatol. 2023 Jan;22(1):111‐118.

The female genitalia comprise functional components crucial for sexual development and an external component—the vulva—that significantly impacts a woman's confidence and is subject to age‐related changes. As described by Triana et al. [8], the vulvar anatomy, from anterior to posterior, includes the mons pubis, labia majora, labia minora, vestibule, external urethral meatus, hymen, and the orifices of Bartholin's, Skene's, and vestibular glands, as well as the perineum. The mons pubis, located superior to the pubic symphysis, is composed of variable amounts of adipose tissue. The labia majora, paired cutaneous folds extending posteriorly from the mons pubis, are wider anteriorly and taper posteriorly, converging with the labia minora at the fourchette. The labia minora, thinner folds located medial to the labia majora, have minimal subcutaneous fat and exhibit asymmetry. The clitoral hood and frenulum constitute the anterior aspect of the labia minora [3, 8, 9](Figure 1).

Within the vaginal vestibule, the external urethral orifice lies approximately 2.5 cm posterior to the clitoral glans and anterior to the vaginal introitus [8]. The vestibular bulbs, located laterally to the vaginal and urethral openings, consist of erectile tissue connected to the urogenital diaphragm by the bulbospongiosus muscle. The clitoris, a crucial female sexual organ, is characterized by exceptional sensory innervation. Its glans, a rounded structure, is situated at the anterior convergence of the labia minora, superior to the urethral and vaginal openings [3, 8]. The clitoral body, measuring approximately 2.5 cm in length, is anchored to the pubic bone by the suspensory ligament, and the glans is formed by the convergence of the labia minora [3] (Figure 1).

### 1.2 Psychological Impact

The International Society of Plastic Surgery (ISAPS) 2023 annual survey has reported a significant increase in female genital cosmetic surgery for vaginal rejuvenation procedures, with a 19.6% increase compared to 2022. Additionally, there was a 14.8% increase in labiaplasty procedures in 2023. Specifically, in Colombia, 2172 vaginal rejuvenation procedures were completed in 2023 [7]. These surgical and noninvasive energy‐based procedures, such as lasers and radiofrequency, are gaining more popularity nowadays.

The societal idealization of specific female genital appearances has influenced perceptions of normalcy [3]. Individual vulvar morphology varies considerably based on age, ethnicity, hormonal status, and personal health history. However, readily available media portrayals, including internet content and pornography, have contributed to the dissemination of unrealistic esthetic standards, [10] potentially leading to anxiety, insecurity, and diminished self‐esteem among women seeking treatment options.

The impact of FGCS on psychological well‐being is significant. Goodman et al. [11], in a prospective controlled study of 120 patients undergoing labiaplasty, clitoral hood reduction, and esthetic vaginal tightening (perineoplasty + “vaginoplasty” or “vaginal rejuvenation”), reported improvements in postoperative body image, genital self‐image, sexual satisfaction, and self‐esteem [11].

### 1.3 Definition of Beauty in the Female Genital Area

Defining esthetic ideals for female external genitalia remains challenging, as there is no universally accepted standard. These ideals can vary significantly based on influences from social media, the fashion industry, and global expectations [3, 4, 10]. Early attempts to define these standards, such as Hodgkinson et al.'s (1984) description favoring smaller labia minora and larger labia majora, remain subjective [12]. More recent classification systems, such as Motakef's 2015 framework, which focuses on labia minora protrusion relative to the labia majora, [13] primarily address surgical considerations and do not directly apply to filler‐based treatments. While some suggest parallels between concepts of symmetry and proportion and the morphology of the *Clitoria ternatea* flower, [3, 6, 9] this association influences perceptions of beauty. Nevertheless, the lack of a standardized aesthetic classification underscores the need for further research to enhance understanding of the diverse preferences and perceptions regarding female genital esthetics.

### 1.4 Overview of Labia Majora Rejuvenation

Esthetic gynecology encompasses a variety of procedures tailored to the patient's preferences and anatomical needs [8]. These procedures can be categorized into surgical techniques (Labiaplasty, Clitoral Hood Reduction, Vaginal Rejuvenation, Labia Majora Augmentation, Hymenoplasty) [3, 8] Nonsurgical Techniques (LASER Treatment for Vaginal Laxity, Radiofrequency [RF], Vaginal Rejuvenation, Vulvar Lightening, Platelet‐Rich Plasma (PRP) Therapy, Hyaluronic Acid [HA] Filler, Lipofilling) [9, 10].

Surgical techniques primarily focus on excess skin of the labia minora or atrophy of the vaginal mucosa [6, 14]. On the other hand, nonsurgical approaches focus more on the cosmetic enhancement of the labia majora, mainly using hyaluronic acid or calcium hydroxyapatite, [6] which have been documented in the literature as an effective method for improving the appearance of external genitalia. A recent study by Dr. Clarissa Lima Vilela and colleagues [6] evaluates the efficacy, tolerability, and safety of using hyaluronic acid (HA) and calcium hydroxyapatite (CaHA) for the treatment of atrophic labia majora, advocating for a personalized approach based on the patient's degree of labial laxity [6].

## 2 Literature Review

This narrative review examines noninvasive labia majora rejuvenation techniques utilizing hybrid fillers. A comprehensive search of the PubMed and Google Scholar databases (2015–2024) was conducted using keywords such as “labia majora,” “rejuvenation,” and “fillers.” After screening titles and abstracts to eliminate duplicates and low‐quality studies and selecting the most recent versions of any updated publications, 10 articles were chosen for in‐depth analysis from an initial pool of 30.

Early reports of injectable treatments for labia majora rejuvenation date back to 2016, when Fasola et al. [15] described the use of hyaluronic acid (HA) in 54 patients. The study reported significant improvements in Global Aesthetic Improvement Scale (GAIS) scores, demonstrating HA's safety and efficacy with minimal complications and reversibility. A 2017 review by Jabbour et al. [4] analyzed nine studies involving 226 patients focusing on vaginal rejuvenation. While fat grafting was the most common technique, HA was utilized in two studies (2–6 mL/session), with no serious adverse events reported [4].

However, potential complications were highlighted by Kwon et al. [16] in 2021, who reported a case of sepsis and necrotizing fasciitis following labia majora filler injection, underscoring the importance of careful technique and material selection. Recent studies have further investigated the use of HA and calcium hydroxyapatite (CaHA) for labia majora rejuvenation. Vilela et al. [6] (2024) compared HA and CaHA, finding significant improvements in volumization and flaccidity at 90 days. HA demonstrated higher patient satisfaction, with 80% reporting excellent improvement compared to 50% for CaHA [6]. Additionally, Ayatollahi et al. [5] (2024) supported HA's safety and efficacy in postmenopausal women using low‐molecular‐weight HA, reporting no complications [5].

To date, a search of PubMed and Google Scholar has revealed no indexed case reports documenting the use of hybrid HA/CaHA fillers for labia majora rejuvenation.

## 3 Case Presentation

**FIGURE 2:**
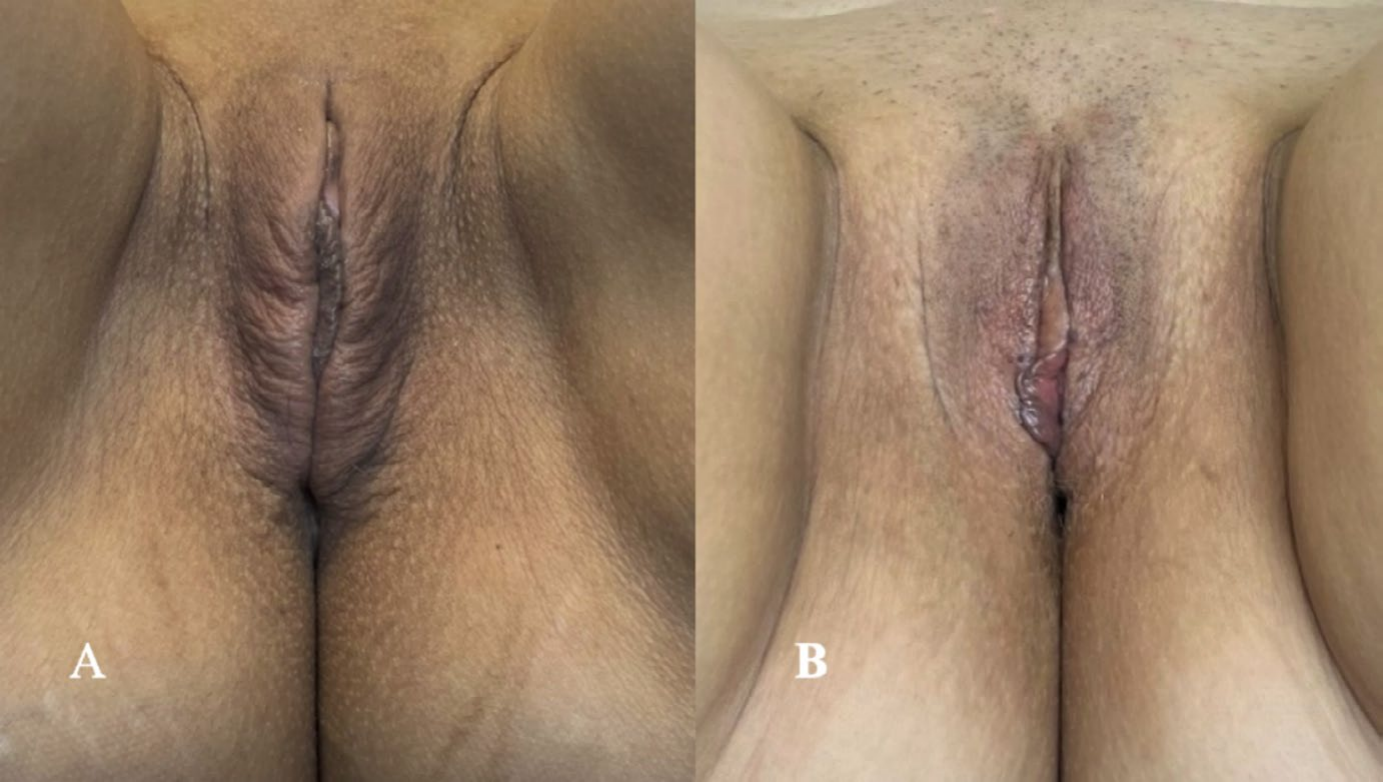
Pretreatment photographs. (A) Patient 1 (Motakef Class I protrusion), exhibiting volume loss, skin laxity, and hyperpigmentation. (B) Patient 2 (Motakef Class II protrusion), demonstrating volume loss and altered pigmentation.

Two patients (ages 47 and 50 years old) from Colombia, seeking nonsurgical treatment, presented with labia major volume loss, skin laxity, altered pigmentation, and asymmetry, resulting in decreased self‐confidence. Written informed consent was obtained for photographic documentation and future publication in scientific journals. The Genital Appearance Satisfaction (GAS) scale questionnaire, where a higher number is associated with dissatisfaction, [17] was applied; the initial number for both patients was 27/33 and 29/33; at physical examination, patients presented labia minora protrusion grade I (patient one) and II (patient two), according to Motakef's scale, and middle sagginess, also presenting an essential loss of volume and hyperpigmentation in the area. (Figure 2) Based on these findings, a hybrid filler approach was implemented.

### 3.1 Hybrid Filler Preparation

A 3 mL mixture of 1.5 mL CaHA (Radiesse, Merz Aesthetics) and 1 mL AH (Belotero Balance, CPM‐B, Merz Pharmaceuticals GmbH) was created by combining and mixing the products 10 times using a Luer‐lock connector plus 0.5 mL of 2% lidocaine without epinephrine. According to vulvar morphology, the estimation of skin laxity is based on the experience of previous authors who describe similar filler techniques [1, 18].

### 3.2 Injection Technique Description

**FIGURE 3:**
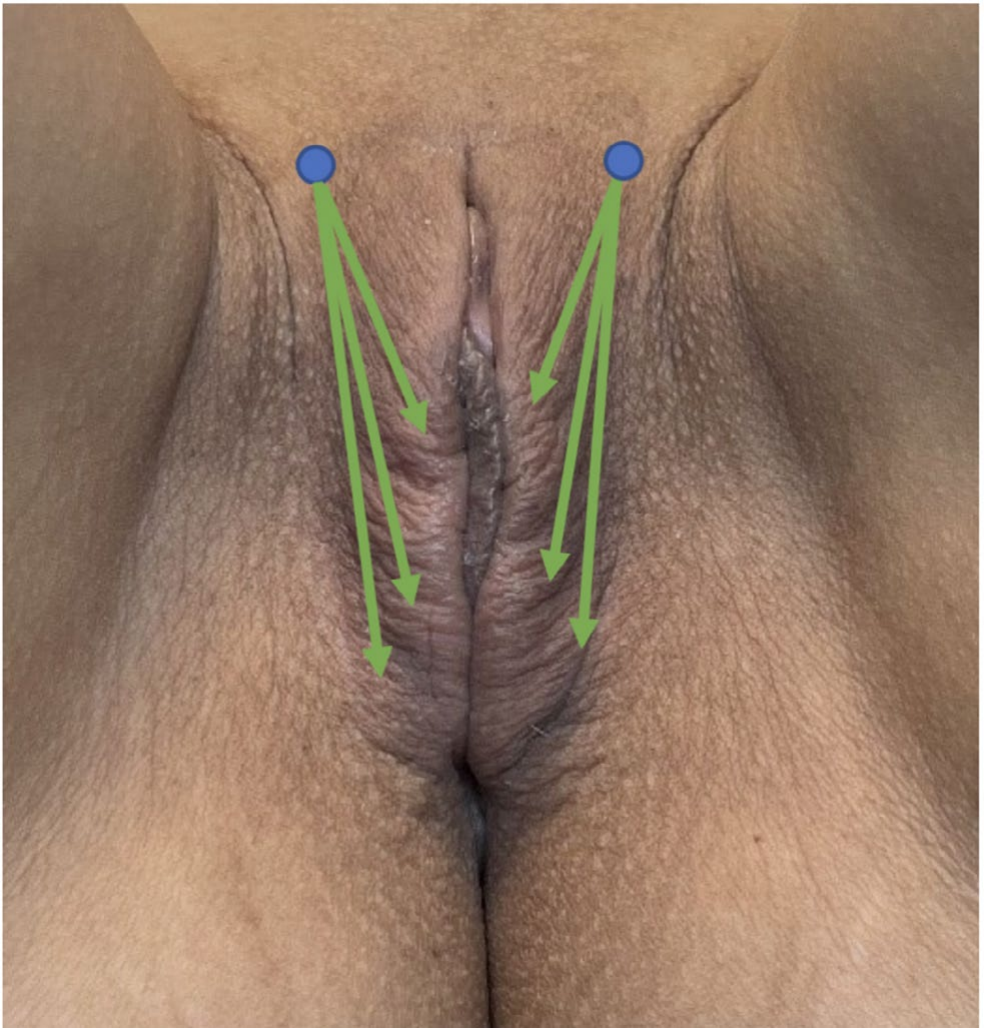
Injection technique. Two 1.5 cm lateral entry points (blue circles) on the mons pubis were used. Hybrid filler (1.5 mL per side) was retroinjected via cannula, guided by green arrows, superficially within the dermis.

**FIGURE 4:**
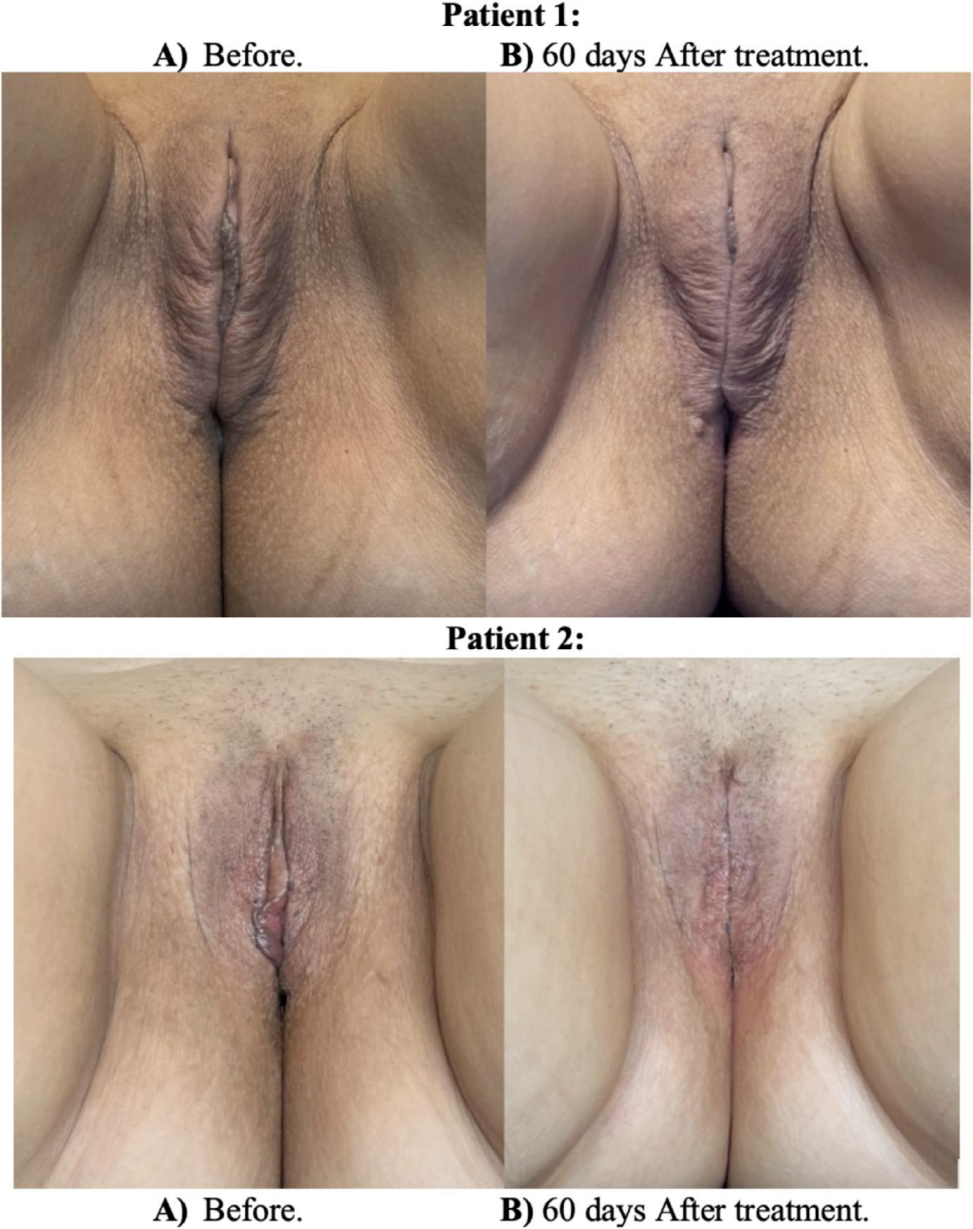
Pre‐ and 60‐day post‐treatment results for two patients (A, B: Patient 1, aged 47; C, D: Patient 2, aged 50). Both patients reported no pain and experienced no complications.

The treatment area was cleansed with chlorhexidine solution, extending from the mons pubis inferiorly and posteriorly to the perineum. Two lateral entry points, approximately 1.5 cm lateral to the clitoris on the mons pubis, were established (Figure 4). At each site, 0.1 mL of 2% lidocaine without epinephrine was administered subcutaneously. A 22G x 50 mm cannula delivered 1.5 mL of the hybrid filler to each labium majora using a retroinjection technique (Figure 3 green arrows). The filler was deposited superficially within the dermis in small increments to ensure even distribution. Postinjection, a gentle massage was applied. A qualified aesthetic practitioner performed all procedures. Post‐treatment monitoring for adverse events and patient instruction regarding reporting any concerns to medical staff was implemented.

A final follow‐up evaluation was conducted 60 days after the procedure, with the GAS scale questionnaire [17] administered pre‐ and post‐treatment. Post‐treatment (60 days), significant improvements in labia majora volume and tone were observed (Figure 4), demonstrating the technique's efficacy.

**FIGURE 5:**
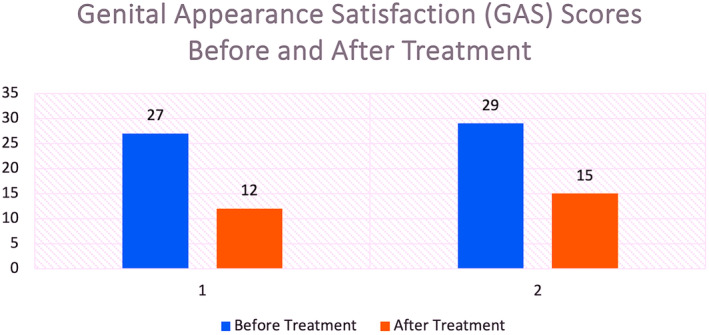
Genital appearance satisfaction (gas) scores: Pre‐ and post‐treatment.

Descriptive statistics were utilized to analyze changes in GAS scores from pre‐ to 60‐day post‐treatment. Table 1 displays the individual patient's pretreatment, post‐treatment, and difference scores. The mean percentage change in GAS score was a 41% decrease. While these preliminary findings, based on a small sample size, require further investigation, the observed improvement suggests the potential clinical benefit of this approach and warrants further study with larger cohorts (Figure 5).

**TABLE 1:** Genital appearance satisfaction (GAS) scores before and after treatment.

Patient	Pretreatment GAS score	Post‐treatment GAS score	Difference	Percentage change
1	27/33	12/33	−15	−43.9%
2	29/33	15/33	−14	−38.1%
**Mean**	**28/33**	**13.5/33**	**−14.5**	**−41%**

*Note:* This preliminary study showed a statistically significant improvement in GAS scores following the treatment (t(1) = 29.00, *p* = 0.022). The mean improvement was 14.50 points (95% CI [8.15, 20.85]). Table 1 presents the individual and mean Genital Appearance Satisfaction (GAS) scores before and after treatment in a pilot study (*n* = 2). A paired‐samples t‐test was conducted to assess the difference between pre‐ and post‐treatment GAS scores. While a statistically significant decrease in GAS scores was observed (t(1) = 29.00, *p* = 0.022). The mean difference between pre‐ and post‐treatment GAS scores was −14.5 points (meaning an average improvement of 14.5 points), with a 95% CI of [8.15, 20.85].Given the small sample size, these findings are preliminary and warrant further investigation with a larger sample to validate these initial observations and confirm the generalizability of the results.

## 4 Discussion

This narrative review highlights the evolving esthetic considerations surrounding the vulva, influenced by media and societal pressures [3]. The labia majora, extending from the mons pubis to the perineum, present a complex anatomical region for treatment due to its proximity to vascular structures [8]. Age‐related changes, hormonal fluctuations, and weight variations contribute to volume loss, laxity, and altered pigmentation [2, 6]. Current treatment modalities primarily involve surgical techniques focused on labia minora reduction [13], while nonsurgical approaches predominantly utilize HA‐based fillers [5] and, more recently, CaHA fillers [6]. However, documented experience with hybrid filler combinations in this area remains limited.

This study explored the potential benefits of a novel hybrid filler combining CaHA and HA. While stand‐alone HA fillers provide immediate volumization with a temporary effect, [5] and CaHA fillers offer long‐lasting results through collagen, elastin, and proteoglycans stimulation, [19] each has limitations. HA's short duration may require repeated treatments. In contrast, CaHA's initial volume effect may diminish as the filler integrates over time and may not be the ideal product to replace volume. Our hybrid approach, involving a homogenous mixture of CaHA and HA in a single syringe, demonstrated promising results. This technique yielded a smooth, creamy gel, potentially enhancing tissue lift compared with CaHA alone and mitigating the early volume reduction sometimes associated with CaHA. The gradual HA degradation and CaHA's collagen‐stimulating properties might result in sustained solmization over time. Fakih‐Gomez et al. have demonstrated the favorable safety profile and aesthetic outcomes of this combined filler approach in other areas (face and hands) [18, 20, 21, 22]. The results in two patients showed improvements in labia majora esthetics, as indicated by an over 15‐point increase in GAS scores. Although these results are promising and suggest the potential benefits of the hybrid filler approach, they are preliminary and require validation through controlled studies with more patients.

Furthermore, given the possible complexity of the procedure, its implementation should only be undertaken by experienced practitioners with extensive expertise in the use of injectable fillers for body and intimate area treatments.

## 5 Conclusion

This narrative review of the literature indicates that vaginal rejuvenation treatments are primarily dominated by surgical procedures aimed at excising excess skin from the labia majora. Noninvasive options mainly include laser treatments designed to enhance skin quality and the application of hyaluronic acid‐based fillers. This underscores the anatomical complexity of the vaginal area, which carries a high risk, thus necessitating treatment by experienced professionals.

We also presented the outcomes observed in two patients who reported significant improvements in the esthetic appearance of their vaginal area following treatment with a hybrid filler, as measured by the Global Aesthetic Improvement Scale (GAIS) score. The results indicate that the procedure is safe, does not result in disabilities or limitations in daily activities, and only minimal pain was reported. The primary aim of the treatment was to restore the esthetics of the vaginal region. A notable finding from our post‐treatment assessments was the significant improvement in the skin color of the treated area, potentially linked to the use of calcium hydroxylapatite (CaHA) and its positive effects on fibroblast activity and enhancements in the extracellular matrix.

According to current literature, the authors posit that combining two types of injectables in a single session may reduce the required volumes of hyaluronic acid, which have been associated with a higher risk of complications. Furthermore, this combination approach simultaneously restores volume, tightens the area, and increases dermal thickness through the properties of CaHA‐based fillers.

While we observed these promising results through comparative photographs, further complementary studies are necessary to validate our findings.

## Author Contributions

A.A.: patient recruitment, treatment, and data collection. A.M.P.: patient treatment, data collection, and manuscript review. V.D.: statistical analysis and data interpretation. L.A.P.: data analysis, interpretation, and manuscript review. D.C.: data cleaning and validation, statistical analysis. L.V.: manuscript drafting and editing, statistical analysis. A.M.A.: manuscript drafting and editing, statistical analysis. All authors reviewed and approved the final manuscript.

## Conflicts of Interest

Authors Andrea Acevedo, Luis Alberto Parra, and Valentina Dicker have received speaker honorariums from Merz Aesthetics Colombia, which owns the licenses for Radiesse and Belotero in Colombia. These honorariums were received before the publication of this paper and were unrelated to the technique of Labia Majora Rejuvenation with Hybrid Filler.

## Data Availability

The data that support the findings of this study are available from the corresponding author upon reasonable request.

## References

[1] F. Tarabini , L. Rozemberg , G. Zapata‐Sudo , et al., “A Novel Hyaluronic Acid Filling Technique for Restoring Volume of the Labia Majora,” Cureus 15, no. 9 (2023): e45728. 37868534 10.7759/cureus.45728 PMC10590245

[2] S. Mohammad , K. S. Joshi , S. Mohammad , et al., “Aesthetic Gynaecology: What Women Want?,” Cureus 15, no. 8 (2023): e44251. 37772220 10.7759/cureus.44251 PMC10523831

[3] A. Jindal , V. Mysore , and J. V. Mysore , “Cosmetic Gynecology‐An Emerging Field for the Dermatologist,” Journal of Cosmetic Dermatology 22, no. 1 (2023): 111–118. 36335587 10.1111/jocd.15484

[4] S. Jabbour , E. Kechichian , B. Hersant , et al., “Labia Majora Augmentation: A Systematic Review of the Literature,” Aesthetic Surgery Journal 37, no. 10 (2017): 1157–1164. 28449124 10.1093/asj/sjx056

[5] A. Ayatollahi , A. Samadi , B. Barikbin , et al., “Efficacy and Tolerability of a Hyaluronic Acid‐Based Extracellular Matrix for Labia Majora Rejuvenation and Augmentation: A Pilot Study,” Cureus 16, no. 4 (2024): e58970. 38800301 10.7759/cureus.58970 PMC11127129

[6] C. L. Vilela , G. E. de Lima Faria , and R. F. Boggio , “Treatment of Atrophy of the Labia Majora: Calcium Hydroxyapatite or Hyaluronic Acid?,” Aesthetic Plastic Surgery 48, no. 3 (2024): 472–477. 37673803 10.1007/s00266-023-03617-3

[7] ISAPS (I.S.O.P.S.) , “International Survey in Aesthetic/Cosmetic Procedures in 2023,” 2023, https://www.isaps.org/media/rxnfqibn/isaps‐global‐survey_2023.pdf.

[8] L. Triana and A. M. Robledo , “Aesthetic Surgery of Female External Genitalia,” Aesthetic Surgery Journal 35, no. 2 (2015): 165–177. 25717117 10.1093/asj/sju020

[9] G. Toplu and D. Altinel , “Genital Beautification and Rejuvenation With Combined Use of Surgical and Non‐Surgical Methods,” Aesthetic Plastic Surgery 45, no. 2 (2021): 758–768. 32997240 10.1007/s00266-020-01980-z

[10] M. Vanaman , J. Bolton , O. Placik , and S. G. Fabi , “Emerging Trends in Nonsurgical Female Genital Rejuvenation,” Dermatologic Surgery 42, no. 9 (2016): 1019–1029. 27153040 10.1097/DSS.0000000000000697

[11] G. J. Goodman , M. R. Magnusson , P. Callan , et al., “Aspiration Before Tissue Filler–An Exercise in Futility and Unsafe Practice,” Aesthetic Surgery Journal 42, no. 1 (2022): 89–101. 33512439 10.1093/asj/sjab036 PMC8670299

[12] D. J. Hodgkinson and G. Hait , “Aesthetic Vaginal Labioplasty,” Plastic and Reconstructive Surgery 74, no. 3 (1984): 414–416. 6473559 10.1097/00006534-198409000-00015

[13] S. Motakef , J. Rodriguez‐Feliz , M. T. Chung , et al., “Vaginal Labiaplasty: Current Practices and a Simplified Classification System for Labial Protrusion,” Plastic and Reconstructive Surgery 135, no. 3 (2015): 774–788. 25719696 10.1097/PRS.0000000000001000

[14] B. Cihantimur and C. Herold , “Genital Beautification: A Concept That Offers More Than Reduction of the Labia Minora,” Aesthetic Plastic Surgery 37, no. 6 (2013): 1128–1133. 24042737 10.1007/s00266-013-0211-4

[15] E. Fasola and R. Gazzola , “Labia Majora Augmentation With Hyaluronic Acid Filler: Technique and Results,” Aesthetic Surgery Journal 36, no. 10 (2016): 1155–1163. 27241363 10.1093/asj/sjw083

[16] B. S. Kwon and J. W. Kim , “Catastrophic Complications From Filler Injection on External Genitalia,” Archives of Plastic Surgery 48, no. 1 (2021): 10–14. 33503739 10.5999/aps.2020.01907 PMC7861980

[17] D. Veale , E. Eshkevari , N. Ellison , L. Cardozo , D. Robinson , and A. Kavouni , “Validation of Genital Appearance Satisfaction Scale and the Cosmetic Procedure Screening Scale for Women Seeking Labiaplasty,” Journal of Psychosomatic Obstetrics and Gynaecology 34, no. 1 (2013): 46–52. 23394414 10.3109/0167482X.2012.756865

[18] N. Fakih‐Gomez , C. Muñoz‐Gonzalez , C. A. Porcar Plana , et al., “Standarized Protocols for Hybrid Filler Application in Aesthetic Medicine,” American Journal of Cosmetic Surgery (2024), 10.1177/07488068241227278.

[19] S. B. Aguilera , A. McCarthy , S. Khalifian , Z. P. Lorenc , K. Goldie , and W. G. Chernoff , “The Role of Calcium Hydroxylapatite (Radiesse) as a Regenerative Aesthetic Treatment: A Narrative Review,” Aesthetic Surgery Journal 43, no. 10 (2023): 1063–1090. 37635437 10.1093/asj/sjad173 PMC11025388

[20] J. v. Loghem , “Use of Calcium Hydroxylapatite for Augmentation of the Labia Majora and Mons Pubis,” 2017.

[21] N. Fakih‐Gomez and J. Kadouch , “Combining Calcium Hydroxylapatite and Hyaluronic Acid Fillers for Aesthetic Indications: Efficacy of an Innovative Hybrid Filler,” Aesthetic Plastic Surgery 46, no. 1 (2022): 373–381, 10.1007/s00266-021-02479-x. 34341855 PMC8831259

[22] G. E. L. Faria , N. Fakih‐Gomez , A. Tartare , et al., “Hand Rejuvenation With Customizable Hybrid Fillers: Premixed Calcium Hydroxyapatite and Hyaluronic Acid,” Aesthetic Plastic Surgery 48, no. 15 (2024): 2887–2894. 38831064 10.1007/s00266-024-04145-4

